# The Social Cognitive Evaluation Battery for Children with Autism: A New Tool for the Assessment of Cognitive and Social Development in Children with Autism Spectrum Disorders

**DOI:** 10.1155/2010/875037

**Published:** 2010-10-24

**Authors:** Eric Thiébaut, Jean-Louis Adrien, Romuald Blanc, Catherine Barthelemy

**Affiliations:** ^1^INTERPSY University of Nancy 2, B.P. 3397, 54015 Nancy, France; ^2^Laboratory of Psychopathology and Health Process, EA 4057, University Paris Descartes, 71, Avenue Edouard Vaillant, 92100 Boulogne-Billancourt, France; ^3^UNIT INSERM 619, University Hospital Center of Tours, Tours, France

## Abstract

The Social Cognitive Evaluation Battery (SCEB) is a new instrument for the psychological evaluation of children with autism. The battery consists of 16 scales that measure different cognitive and socioemotional functions. This study reports the results of a reliability analysis and some elements of validation. Analyses are based on the observed performance of 100 children with autism and a convenience sample of 35 normal children. Validation is based on the examination of the structure of the relations between the 16 scale scores of the SCEB, their relations with other measurements, the correspondence between the theoretical developmental ages, and the observed chronological ages and the SCEB's sensitivity to specific disorders. The results show that this new instrument is useful and relevant for the psychological assessment of children with autism.

## 1. Introduction

In order to develop a behavioral intervention program for children with autism centered on cognitive and socio-emotional abilities, it is necessary to perform specific developmental assessments of these abilities. While there are many instruments for high-level children with autism (intelligence, memory and adaptive scales, executive function tests, and theory of mind tests), there are few validated instruments that specifically assess the psychological development of low-level or very young children with autism. In fact, for the autistic population with mental retardation and a developmental level of under two years of age, it is still necessary to perform an overall examination of the abilities known to develop during the first two years of life. While one could use infant developmental tests that involve normative data on a reference population, such as the Bayley Infant scales [1] or, for a French population, the Brunet-Lézine-Revised scales [2], their contents and procedural standards are not sufficiently adapted to children with autism. In fact, the available tools do not allow a deep clinical analysis of pervasive impairments in the psychological development of children with autism. The Psycho-Educational Profile (PEP 3) [3] specifically assesses the psycho-educative development of children with autism, but it has not been standardized for French populations. Moreover, various cognitive and socio-emotional domains such as object permanence, symbolic play, and self-image are not specifically explored for ages of 4 to 24 months. There are also some instruments for the developmental assessment of children with pervasive developmental disorders that are domain specific: The Ugziris-Hunt scales[4] focus on infant cognitive development; the Early Social Communication Scales [5, 6], Mac Arthur communicative development inventory [7], SCATA [8] and the Communication and Symbolic Behaviour Scales [9] focus on socio-communicative development or Reynell [10] whose focus is language development. However, these specific instruments do allow investigating cognitive, emotional, and communicative developmental domains in a single evaluation. Moreover, while the Vineland scales can jointly assess communicative skills, socialization, autonomy, and motricity of children with developmental disorders whose developmental ages are less than 24 months, the assessment uses parents' reports and not direct observation of the children. In fact, children with autism, whose development level is less than 24 months, are characterized by anxiety, hyperactivity, and many attention disorders. Thus, the examiner must stimulate the children in order to obtain responses to tasks, but also adjust his behavior in order to take into account their attention and communication disorders. Therefore, a single instrument for assessing the overall psychological development involving the various developmental domains known to be disturbed in autism would be helpful to psychologists. If the examiner wanted to assess a particular developmental domain more thoroughly, he could use one of the specific instruments. Moreover, there is real difficulty in using several different instruments relative to the domains of cognitive communicative, and language development; their standardization and normative data are different. A child with autism examined with these three instruments would be compared to three different normative reference samples. The SCEB provides a single and pertinent instrument for the developmental assessment of very mentally retarded or young children with autism, so overcomes the limits of using multiple instruments. 

The Social Cognitive Evaluation Battery** (**SCEB) [11] covers 16 functional abilities; it aims to addresses the clinical needs of psychologists, by contributing to the definition of a personal therapeutic and psycho-educational project adapted to each child [4] and to further research on autism.

### 1.1. Objective

This paper presents the first psychometric analysis of SCEB, an evaluation tool that is useful for both for clinical purposes and research. The analysis can help users decide whether the SCEB is suitable for their purpose.

### 1.2. Description and Origins of the SCEB Scales

The SCEB integrates several models of development including Piaget's and Fisher's [12]. Fisher broadens the Piagetian model by including a hierarchical organization of cognitive and social capacities. Integrating models of development into the SCEB means that the items of the instrument can be associated with a given level of development.

The SCEB covers two general domains, cognition and the socio-emotional domain. The cognitive domain is defined by seven scales of ability: symbolic play, object relation schemata, operational causality, means ends, spatial relations, objects permanence, and self-image. The socio-emotional domain is defined by nine abilities: social interaction, behavior regulation, joint attention, expressive language, comprehensive language, vocal imitation, gestural imitation, affective relation, and emotional expression. 

The first six scales of the cognitive domain are based on the *Infant Psychological Development Scale* [13] and the *Symbolic Play Test* [14]. The self-image scale was developed from the works of lewis & Brooks-Gunn [15] and Berthental & Fisher [12] on self-recognition in young children. The communication scale of Seibert, Hogan & Mundy [5], inspired our scales of socioemotional abilities, communication, behavior regulation, social interaction, and joint attention. The scales evaluating expressive and comprehensive language originate from the tests used in the Piagetian model [16] and from the normative inventories [17]. The evaluation of the development of affective relations is based on the object index [18] and the evaluation of emotional expression on the scales of socioaffective development and behavior [19–21]. As for cognition, we test each of its sub-scales. For object permanence, we assess the capacity of the child to represent a nonvisible object (the examiner hides a marble in a box—the child lifts the box). For means ends, the examiner observes the various means that the child uses to reach an objective (the examiner puts a cloth in a cylinder and the child pulls it out with a toy rake). For operational causality, the way in which the child establishes causal links between his own actions and the various objects he uses is observed (the examiner presents a mechanical toy and sets it going—the child takes the toy and activates its mechanism). For spatial relations, the examiner notes how the child establishes links of proximity, superposition, juxtaposition, fitting, and so forth, (the examiner provides rings to be piled up—the child stacks the rings according to their sizes). With respect to the object relation schemata, the examiner observes the capacity of the child to produce and combine several schemata (the examiner presents a toy—the child can use it in many ways). For the evaluation of self-image, a mirror is used (the nose of the child is marked, the examiner presents the child with a mirror—the child tries to wipe off the mark). 

Social and emotional behaviors are also assessed. We assess the child's ability to communicate and regulate behavior (the examiner gives the child an instruction—the child responds to complex orders), to engage in social interactions (the examiner presents an object—the child takes it and invites the adult to play with him) and to demonstrate joint attention (the examiner points at an object—the child looks at the indicated object). Also assessed are expressive language (the child comments on his actions), comprehensive language (the child understands sentences of several words), vocal imitation (the child repeats a sentence composed with new words), gesture imitation (the child immediately imitates gestures), affective relations (the child cries and is anxious when separated from his parents) and emotional expression (expressions of joy and anxiety). 

We evaluated the relevance and the adequacy of SCEB scores by examining several of their properties. We examined whether the scores are independent of the examiners, interrater reliability. Two examiners observing the same child should provide the same results. We addressed the question of the accuracy of the scores, reliability. Unreliable measurements prevent forming useful hypotheses that can guide a therapeutic strategy. The constructs considered by the SCEB belong to two broad but distinct domains of mental life, cognitive capacities, and socio-emotional abilities. The fit between empirical data and this theoretical conception was assessed through a descriptive and structural analysis. We provided an argument for external validity by comparing the scores of the SCEB to the Brunet-Lezine scales (a French adaptation of Gesell scales that provide a developmental quotient). We expected the correlations to be positive and moderate because the Brunet-Lézine scales are not well adapted to children with autistic disorders and they consider fewer behavioral domains than does the SCEB. Because some items of the SCEB are based on theories of development, their scores also represent a developmental age that is referenced to typical children. We examined the agreement between typical children's theoretical development ages given by the SCEB and their chronological ages, but not by statistical tests because the sample size was too small. However, the assessment is important because it can guide subsequent revisions of the SCEB. Although the SCEB is not intended to differentiate children according to the nature of their developmental disorder, we present some results on the sensitivity of the SCEB to distinguish between developmental disorders. We compare data published by Adrien [4, 22] with data produced for this study, although the results are precarious because of small group size. The results for differential sensitivity of the SCEB scores are compared to those published by Adrien [4], amongst others. In the data published by Adrien, we are interested in the differences studied in the development profiles of two small groups of children paired according to their developmental ages (7 Down syndrome children and 7 children with autism). It appears that in the children with autism the lowest performance occurs in the nine socio-emotional spheres and communicative spheres. In the children with mental retardation, the lowest performance occurs in language (production and comprehension) and imitation (spoken and gestural). The children with autism displayed very heterogeneous profiles, unlike the children with mental retardation.

## 2. Method

### 2.1. Material

The items of SCEB are selected from existing instruments, thus, they have already been tested. Each item was chosen by a committee of experts in the development of young children according to whether it was clearly linked to the construct it was supposed to represent and the degree to which it exclusively represented one of the four levels of development considered within the SCEB.

### 2.2. Procedure

The child is asked to follow the psychologist into an examination room equipped with a video camera; video recordings are mainly used for the study of inter-rater reliability. The SCEB lasts for between 40 and 50 minutes on average. Coding takes 15 minutes. The SCEB procedure unfolds over three stages: behavior coding, developmental coding, and developmental profiling.

#### 2.2.1. Behavior Coding

The child and the examiner, who has SCEB materials at hand, are situated in a simple environment designed to make the child feel calm and safe, and, therefore, receptive. Following a period of familiarization, the examiner proposes a series of activities and games (building blocks, dolls, a toy snack bar, toy cars, etc.) in accordance with the pace, attention, and interests of the child. The examiner completes an observation grid composed of 188 items of the 16 SCEB subscales during the examination, immediately afterwards, and while watching the video recording. Entries in the grid are binary, depending on the presence or absence of the behavior mentioned in the item. This initial stage of the measuring behavior with the observation grid provides the most objective data for evaluating the child's level of development.

#### 2.2.2. Developmental Coding

The examiner then fills in the 128 items evaluating the developmental levels of the abilities as a way to evaluate level of aptitude. The items are scored on a three-point scale: 0 = failure, 1 = behavior in emergence or completed with the help of the examiner, and 2 = stable behavior completed without help. The items themselves are organized according to the four levels of development: level 1: 4–8 months; level 2: 8–12 months; level 3: 12–18 months and level 4: 18–24 months. Each of the 16 scales is also ranked according to the four levels.  Table 1 give examples of items taken from the 16 developmental level scales of the SCEB.

#### 2.2.3. Developmental Profiling

**Figure 1:**
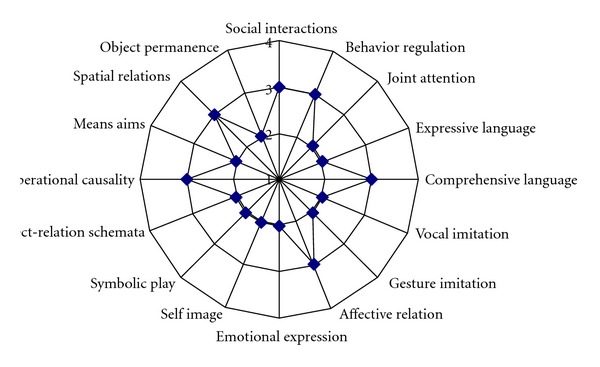
Illustration of the developmental profile of an 8-year old autistic child.

Using the scores on the items, the examiner defines the child's development level and establishes his or her profile (Figure 1). 

Both the psychomotor development scales of Brunet-Lézine [2] and of the SCEB were used for this study. The Brunet-Lezine scales, a French adaptation of the Gesell scale [23], permit an investigation of four areas: language, posture-motor abilities, oculo-manual abilities, and sociability.

### 2.3. Participants

Analysis and psychometric data are based on different samples. Reliability analysis and internal and external relationships of scores are based on data of the 100 autistic children (23 girls and 77 boys) aged between 28 months and 14 years, 6 months (*M* = 5 years 9 months; *SD* = 2 years, 3 months) (Table 2).

The children were recruited from several specialized centers between 1998 and 2002. Most of them attended the Day Unit of the Department of Child Psychiatry of the *Centre Hospitalier Universitaire* of Tours (France). Their diagnosis was carried out at the centers at the request of doctors or families. All children included in the study had been diagnosed as autistic by a psychiatrist after a quantitative assessment using the French adaptation of the *Childhood Autism Rating Scale *(CARS) [24] and the *Diagnostic and Statistical Manual of Mental Disorders*(DSM-IV) criteria [25] and had CARS scores over 30 (see Table 3). There were no children with Asperger's syndrome in the sample.

The children's parents were informed of the aim of the research, which is considered to be of “direct personal benefit” to each child, and their consent was requested. The Ethical Committee of Hospital Center of Tours (France) approved the research. Psychologists trained in using the SCEB and who had good clinical and practical experience with children having pervasive developmental disorders performed the assessments. 

The descriptive analysis of the agreement between the children's theoretical developmental ages and their chronological ages was carried out on data obtained from a small sample of 35 typical children composed of 22 boys and 13 girls aged between 5 and 27 months (*M* = 14 months, *SD* = 6 months). This convenience sample was recruited from the environments of psychologists involved in the study.

Preliminary results on the differential sensitivity of the SCEB are based on data published by Adrien [4, 14]. His data are compared with paired sub-samples from the main sample of 100 autistic children.

## 3. Results

### 3.1. Measurement Reliability

We assessed reliability of the SCEB measurements in terms of inter-rater reliability and internal consistency. To assess inter-rater reliability, 24 of the 100 video recordings were randomly sampled. One psychologist, from a pool of eight psychologists, viewed each video recording. All eight members of the pool were familiar with the SCEB. Their viewing of the video recordings and the actual SCEB behavioral and developmental assessments were independent. We found that if the examiner had not completed the behavioral ratings and subsequently did complete the development scales, then the inter-rater reliability was not satisfactory. It appeared that repeated use of the SCEB could lead the users to overrate the objectivity of their judgment regarding the level of a child's development; they were confident that their objectivity would not be affected if they reduced the length of psychological assessment, by omitting the stage of behavior coding. The observation helps us to define the best practices for administering the SCEB. Thus, for this report, we used only data from examiners who started their evaluations with the behavioral scales and then proceeded to the evaluation on the scales of level of development. 

The findings concerning the final scores of developmental levels. The inter-rater reliability expressed by an intraclass correlation agreement [26] with randomly selected examiner pairs gave an average value of  .83 (Table 4). Due to the small size of the sample, a confidence interval of intraclass correlation is also reported. The intraclass correlations reached satisfactory values, with the exception of the emotional expression subscale (.58) which, although statistically significant, was rather low.

For the scales concerning behavior coding: the internal consistency index is represented by the coefficient KR20 [27] for dichotomous data. Although the Cronbach *α* [28] is not theoretically suitable for dichotomous data, we have observed that the value of its coefficient index and that of KR20 were very similar for the behavior coding data. We report the Cronbach *α* for the levels of development scores.

The values of the reliability coefficients of the subscales for behavior coding are satisfactory overall, with the exception of the subscales “emotional expression” and “object relation schemata”. (see Table 4). In the case of the scales of developmental levels, the reliability ranged from satisfactory to excellent, although the “operational causality” scale did not yield very satisfactory results.

### 3.2. Structure of the Relations between SCEB Subscale Scores

**Figure 2:**
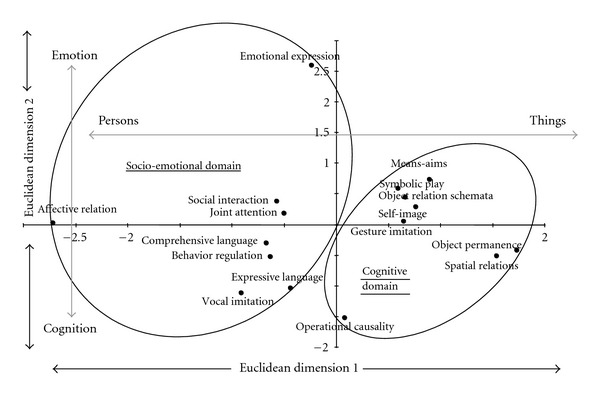
Configuration of SCEB Scores: Plans 1 & 2.

We analyzed the structure of the relations between the SCEB scores for development level with a multidimensional scaling model (MDS) [29]. The MDS provides a spatial representation (Euclidean space) of the relation between the SCEB scales. From a nontechnical point of view, multidimensional scaling (MDS) provides a visual representation of the pattern of proximities among a set of objects. Unlike a numerical presentation, the spatial format facilitates the general understanding of the organization of relations between the scales of the SCEB. The MDS maps are also more robust and parsimonious than the results of factor analysis. Two indexes are usually proposed for the fit between the MDS maps and the matrix input. Stress Formula 1 represents the degree of correspondence between the distances between points implied by the MDS map and the matrix input. A stress value of zero is a perfect fit, but it is not necessary for an MDS map to have zero stress in order to be useful. A certain amount of distortion is acceptable. The RSQ index gives the proportion of common variance between the spatial distance between points and the matrix input. The analysis was conducted on a matrix of proximity between scores of levels of development represented by a correlation coefficient. A three-dimensional solution (Stress = .095; RSQ = .939) is necessary for a good fit. Nevertheless, we propose a two-dimensional solution that is simpler, although less representative of data (Stress = .20; RSQ = .81), because it seems sufficient to show an empirical organization that is compatible with a theoretical distinction between the domains of development (Figure 2). The spatial representation readily differentiates two-scale clusters, the cognitive and the socio-emotional domains. The combination of a dimension opposing “persons” to “things” with a dimension opposing “emotional” to “cognitive” functions can explain the differentiation of the two scale clusters. 

### 3.3. Relations between the SCEB and Brunet-Lezine Psychomotor Development Scales

The correlations between the SCEB and the Brunet-Lézine developmental level scores provide an argument for convergent validity because both tools measure developmental domains. However, we expected only moderate correlations because the two tools do not measure exactly the same components of a domain and are not equally adapted for children with autistic disorders. The correlations between the developmental ages, in months, of the SCEB and the Brunet-Lézine are, for various subscales, Posture, range = .23–.64 (mean = .49), Coordination, range = .29–.79 (mean = .63), Language, range  = .342013.82 (mean = .61) and Sociability, range  = .34–.71 (mean = .61). These values show strong links between the SCEB and the Brunet-Lézine scores; their moderately high values are a good indicator of the convergent validity of the SCEB subscale scores.

### 3.4. Agreement between Children's Theoretical Development Ages and Their Chronological Ages

A comparison of a child's chronological age and theoretical age as predicted by the SCEB was carried out on data obtained from a convenience sample of 35 typical children. The children's developmental scores from the SCEB were placed in one of four developmental levels, 4–8, 8–12, 12–18 or 28–24 months, and the mean chronological age of the children in each level was computed (Table 5). 

The instances where the mean chronological age falls outside the range of the developmental levels are few in number and generally small in magnitude. The subscales showing the greatest discrepancies are Expressive Language, Vocal Imitation, Gesture Imitation, and Symbolic Play. Overall, agreement is satisfactory, but the small sample size does not guarantee the stability of correlation. The findings should be seen as provisional. 

### 3.5. Differential Sensitivity of the SCEB

Although the aim of SCEB is not to differentiate groups of children according to the nature of their developmental disorders better than other instruments, we nevertheless examined the possibility that it does so by comparing published data with those of our sample of 100 autistic children.Adrien [22] published data on two groups of children, average age 5 years: 18 children with mental retardation and 43 children with autism. A cluster analysis (average method based on squared Euclidean distance) shows that 2/3rds of the children with mental retardation formed a coherent class, suggesting that they can be characterized by a distinct profile. The development levels of the children with mental retardation were systematically and significantly higher than the levels of the children with autism, although not for all subscales of the SCEB. The largest differences between the groups were found on the interaction and joint attention scales and the smallest on the means ends, spatial relations, and object permanence scales. Furthermore, Adrien [22] observed that children with autism display more diversity in the way that they develop than do children with mental retardation. If this greater diversity is peculiar to children with autism as opposed to ordinary children, it is significantly greater among multihandicapped children [4]. No significant correlations could be found between the average profile of the levels of development recorded with the SCEB on a sample of 20 severely multi-handicapped children (mean  age = 8) and the profiles of our sample of 100 children with autism. In contrast to our subjects with autism, the highest levels of development for the multi-handicapped children are in Affective relation, Behavior regulation, Joint attention, and Social interaction, while no development is found for Vocal imitation. We compared the data published by Adrien [22] on 11 children with mental retardation to the data from 29 of our 100 subjects matched on gender and chronological age. The CARS, Brunet-Lézine and SCEB scores of the mentally retarded children were available to us. To quantify the differential sensitivity of the scores, we used a logistic regression model with “belonging to the group” as dependent variable and the scores acquired with the three instruments as independent variable. The Cox & Snell R square [30] is similar for the scores obtained with CARS (*R* ^2^ = .69, *X* ^2^  (1) = 47.05, df, *P* < .001) and those with SCEB (*R* ^2^ = .69, *X* ^2^(1) = 47.05, df, *P* < .001). The discriminative power of the scores obtained with Brunet-Lézine appears lower (*R* ^2^ = .48, *X* ^2^(4) = 25.84, *P* < .001). The SCEB has the advantage of providing detailed representations of developmental profiles. Recall, however, that these results are probably unstable because of the small sample sizes.

## 4. Discussion

The psychometric analyses conducted on data obtained from the SCEB provide information on the various properties of the measures and their meaning. The analyses provide information on the objectivity and reliability of the measures, the structure of the relations between the subscale scores, the convergent validity of SCEB scores, as well as a preliminary indication concerning score interpretation in terms of developmental age and differential sensitivity. Regarding objectivity, the present findings demonstrate satisfactory inter-rater reliability, with the exception of the emotional expression subscale. This exception is not surprising given the difficulty of the examiner's task, which is to find observable indications of internal emotional states such as anxiety and sadness. The reliability of the measures expressed by an internal consistency index is satisfactory for the scales of developmental levels, with the exception of the operational causality scale. So the examiners were advised not to consider the score on this scale with the same level of confidence as that of the other subscales. An index of convergent validity was obtained by correlating the SCEB developmental level scores and the developmental ages measured by the Brunet-Lézine instrument. The level of these correlations constitutes an important indicator of the validity of the SCEB subscale scores; the correlations are weakest for the affective relations and emotional expression dimensions. The SCEB aims at measuring the cognitive and socio-affective functions of children with autism, and we observed a strong link with the measures from the Brunet-Lézine psychomotor development scales. The present analysis shows that the SCEB measures can represent those obtained by the Brunet-Lézine psychomotor development scales, while the converse is not true. The SCEB extends the investigation to behavioral domains not considered by the Brunet-Lézine psychomotor development scales; essentially domains concerned with components of the socio-affective sphere. Moreover, the SCEB yields indexes that are more specific. The variety of component scales of the SCEB renders it differentially sensitive to the children's developmental dysfunctions, as demonstrated by the data obtained by a number of comparative studies. Because the essential psychometric qualities of the SCEB have been found to be satisfactory, it may be administered with confidence as part of the psychological examination of children with autism. Because the SCEB extends the investigation to behavioral domains neglected by other instruments, it can contribute new insights regarding children with autism and to the development of individualized educational and therapeutic interventions. Moreover, a series of successive assessments of a particular child with autistic cognitive and socio-emotional development allows his education intervention program to be adapted and his developmental trajectory identified.

The agreement between the children's theoretical development ages predicted by the SCEB and their chronological ages is, at this point, uncertain. Investigations with larger and more representative groups of participants will continue. The few weaknesses of the SCEB revealed by this study can help to define guidelines for its future development and improvement. This new Social Cognitive Evaluation Battery offers a pertinent assessment of cognitive and socio-emotional development in mentally retarded children with autism or young children with autism. Currently, the SCEB is in the process of being culturally and linguistically adapted for use with American, Lebanese, Algerian, Italian, Spanish, and Brazilian populations, with the aid of local psychologists and physicians specialized in cognitive and socio-emotional developmental disorders in children with autism.

## Figures and Tables

**Table 1:** Examples of items taken from the 16 developmental level scales of the SCEB.

Subscales	Examples of items
*Social interaction*	He or she knows how to solicit the adult (by gesture or verbally) to take part or follow with in a parlor game with or without an object
*Behavior regulation*	He responds to a simple order
*Joint attention*	When an adult points to an object, he looks directly at the object indicated
*Expressive language*	He can comment on his own actions
*Comprehensive language*	He understands sentences of two familiar words in context.
*Vocal imitation*	He can imitate familiar noises (car engine, horn, machines etc)
*Gesture imitation*	He can immediately imitate a gesture that he knows
*Affective relation*	He recognizes and differentiates his parents
*Emotional expression*	He smiles at the appearance of an object he wants
*Self-image*	He can name and indicate the parts of his face
*Symbolic play*	He can perform playful actions with unrelated objects (e.g., simulating phoning or a flying plane with a pen.)
*Object relation schemata*	He handles objects in an exploratory way (turning over, shaking, scratching, hitting etc.)
*Operational causality*	When a mechanical toy stops working, the child looks at it impatiently
*Means-ends*	He uses the handle of a toy rake to pull a cloth out of a cylinder
*Spatial relations*	He can fit objects of different shapes into each other
*Object permanence*	An object hidden under one box is moved in view of the child and hidden under another box, the child immediately finds the object under the second box

**Table 2:** Age distribution of subjects with Autism.

*Age class in year*	2-3	>3–5	>5–7	>7–9	>9–11	>11–13	>13–14
*Frequency *	6	40	29	15	8	1	1

**Table 3:** Descriptive statistics of CARS scores.

	Mean	SD	Range
*Cars*	39.4	5.56	30.5–60.0

**Table 4:** Subscale Internal Consistency for Behavior Observation Grids, internal consistency and intraclass correlation agreement for Developmental Level Scales.

Subscales	Internal consistency (*n* = 100)				Inter-rater reliability (*n* = 24)	
	Behavior coding		Developmental coding		Developmental coding	
	KR20	Number of items	Cronbach *α*	Number of items	Intraclass correlation agreement	CCI 95%

*Socio-affective domain*						
*Social interactions *	.83	10	.80	9	.84	.68–1.00
*Behavior regulation *	.83	15	.74	8	.90	.79–1.00
*Joint attention *	.81	9	.87	10	.85	.75–.95
*Expressive language *	.91	11	.85	7	.94	.88–1,00
* Comprehensive language*	.85	8	.90	9	.84	.75–.93
* Vocal imitation*	.84	15	.84	6	.80	.68–.92
* Gesture imitation*	.75	12	.79	6	.89	.80–.98
* Affective relation*	.73	9	.80	12	.82	.70–.94
* Emotional expression*	.59	8	.73	11	.58	.38–.78
*Cognitive domain*						
* Self image*	.85	18	.86	12	.76	.59–.93
* Symbolic play*	.74	7	.71	5	.89	.81–.97
*Object-relation schemata *	.67	6	.82	5	.77	.65–.89
* Operational causality*	*.80*	*8*	*.51*	*6*	*.83*	*.68–.98*
* Means aims*	.84	16	.80	9	.89	.79–.99
* Spatial relations*	.89	14	.93	8	.84	.75–.93
* Object permanence*	.94	19	.81	5	.81	.68–.94

**Table 5:** Agreement between theoretical and observed age.

Subscales	Theoretical developmental age in months			
	4–8	8–12	12–18	18–24
*Social interaction*	5.80^a^	7.33	4.36	21.85
	1.79^b^	1.86	2.91	2.64
*Behavior regulation*	6.00	10.33	13.63	21.40
	1.41	1.53	2.39	2.72
*Joint attention*	6.00	11.29	15.80	21.64
	1.41	1.60	2.17	2.65
*Expressive language*	6.64	14.00	14.80	21.85
	1.91	3.35	2.59	2.64
*Comprehensive language *	6.30	11.00	13.00	20.71
	1.64	1.41	2.28	3.26
*Vocal imitation *	6.33	12.64	19.20	22.30
	1.94	3.38	2.68	2.50
*Gesture imitation *	5.71	12.11	17.60	22.00
	.95	2.37	2.61	2.59
*Affective relation *	5.00	7.75	14.50	21.50
	.0	2.83	3.16	2.85
*Emotional expression *	5.50	9.78	15.71	21.85
	.84	2.68	2.69	2.64
*Self-image*	6.25	8.00	14.73	21.46
	1.75	2.65	3.38	3.36
*Symbolic play*	6.64	14.38	18.90	23.00
	1.91	3.02	3.98	2.68
*Object-relation schemata *	6.14	8.67	17.76	23.60
	1.57	2.66	3.73	2.68
*Operational causality *	5.71	8.25	13.38	20.94
	.95	2.22	2.92	3.02
*Means end*	6.00	10.25	15.91	22.27
	1.41	1.25	3.02	2.57
*Spatial relations *	6.00	8.30	13.00	20.50
	2.0	2.57	.50	3.28
*Object permanence*	6.00	12.67	15.08	22.56
	1.41	4.04	3.59	2.35

^a^Mean of observed age.

^b^SD of observed age.
